# Evaluation of the Effect of the Loading Time on the Microtensile Bond Strength of Various Restorative Materials Bonded to Silver Diamine Fluoride-Treated Demineralized Dentin

**DOI:** 10.3390/ma15134424

**Published:** 2022-06-23

**Authors:** Mohammed M. Aldosari, Fares S. Al-Sehaibany

**Affiliations:** Department of Pediatric Dentistry and Orthodontics, College of Dentistry, King Saud University, Riyadh 11545, Saudi Arabia; aldosarimd@gmail.com

**Keywords:** microtensile bond strength, silver diamine fluoride, restorative materials

## Abstract

The aim of this study was to evaluate the effect of immediate versus delayed loading times on the microtensile bond strength (μTBS) of restorative materials, including resin-based composite (RBC), resin-modified glass ionomer cement (RMGIC) and glass ionomer cement (GIC), that were bonded to silver diamine fluoride (SDF)-treated demineralized dentin. Ninety caries-free extracted premolar teeth were assigned to three groups (*n* = 30) loaded with RBC, RMGIC and GIC restorative materials. Each group was further divided into three subgroups (*n* = 10): subgroup A (control specimens), immediate loading of the restorative material on sound dentin; subgroup B, demineralized dentin, SDF treatment and immediate loading of the restorative material; and subgroup C, demineralized dentin, SDF treatment and restorative material loading a week later. One-way ANOVA and Tukey’s post-hoc tests were performed to compare the μTBS values. The RBC exhibited the highest µTBS, followed by RMGIC and GIC. Multiple comparisons showed an increase in the µTBS in the delayed loading groups irrespective of the restorative material used. The majority of the failure modes were adhesive. Delayed loading of RMGIC for 1 week after SDF application showed significantly higher µTBS than that of immediate loading.

## 1. Introduction

Despite the numerous innovations and advancements that occur with dental materials every year, dental caries has remained a global health problem that challenges dentists in the treatment of affected individuals [1]. The use of topical SDF as an anticaries agent was first promoted in Japan in 1969 [2]. Due to its cost-effectiveness, ease of use, pain-free application and effective outcome in arresting caries progression compared to fluoride varnish and other fluoride-releasing compounds [3,4], the use of SDF is gaining popularity, and SDF is ideal for the high caries risk groups as a nontraumatic and non-aerosol-producing treatment modality [5,6]. Moreover, the application of SDF as a proactive anticaries pretreatment material followed by adhesive minimally invasive restorations in cavitated lesions may help in the prevention of recurrent caries [7,8]. SDF use is not only limited to preventing caries in children [9] and older adults [10] but also as a desensitizing or cavity cleaning agent [11] and irrigant in endodontic treatment [12].

The scientific literature on the exact mode of action of SDF is still unclear. However, the anticaries potential of SDF is evident in its black staining ability, which is indicative of active caries arrest [13]. Potassium iodide (KI) has been used following SDF application to minimize discoloration [14]. Another alternative approach that follows SDF application is restoration with tooth-colored restorative materials, which not only covers the black stains caused by SDF but also restores the loss of tooth structure that is a result of cavitation and improves esthetics and masticatory function [15]. Promising results were obtained when tooth-colored restorations were added to SDF-treated teeth, and as a treatment modality for caries management in children, a wider parental acceptance was obtained with this treatment [16].

The components in SDF play important roles in arresting caries. Silver in SDF helps to stop the degradation of collagen along and provides strong antibacterial effects when it reacts with hydroxyapatite; in addition, fluoride is well known for its remineralizing actions that enhance the mineral content, which in turn hardens the tooth structure weakened by caries [17]. SDF shows a reservoir effect that enables silver on dead cariogenic bacteria to be reactivated and produce a sustained antibacterial effect [13].

Numerous studies have reported conflicting evidence on the bond strength of restorative materials that were subjected to anticaries treatment with SDF or SDF/KI. Some research found no effect on the bond strength of restorative materials such as resin-based composite (RBC) [18], resin-modified glass ionomer cement (RMGIC) [19] or glass ionomer cement (GIC) [20], while others suggested that a precautionary rinse should be performed following SDF application and prior to immediate bonding of direct restorations [13,21].

Heterogenous materials such as anticaries agents and restorative materials have a tendency to interact when used for the purpose of remineralization of affected dentin and eventual reconstruction of lost tooth structure, respectively. The adhesiveness and bond strength of restorative materials were found to be altered as a result of caries-affected dentin and are not the same as adhesion to sound dentin [22]. Likewise, any materials applied to the dentin prior to the highly sensitive restorative technique may adversely compromise the bonding of the restorative material [23]. Therefore, the aim of the present study was to evaluate the effect of immediate versus delayed loading time on the microtensile bond strength (μTBS) of restorative materials, including RBC, RMGIC and GIC restorative materials bonded to SDF-treated demineralized dentin. The null hypothesis tested in the present study was that demineralized dentin treated with SDF did not affect the μTBS values of the restorative materials with regard to the time of loading.

## 2. Materials and Methods

### 2.1. Specimens and Sampling Technique

The sample of this study consisted of 90 caries-free premolar teeth, extracted for orthodontic reasons, collected from dental clinics in Riyadh, Saudi Arabia. The inclusion criterion was sound teeth. The exclusion criteria were teeth with restoration or caries. To remove debris, blood and plaque, the teeth were thoroughly cleaned under running tap water with the help of a soft toothbrush and then placed in freshly prepared 0.5% chloramine-T solution and stored at 4–7 °C until further use. The sample was randomly split into three groups (*n* = 30) using a simple random sampling technique as follows: the first group (I) was loaded with RBC (Neo Spectra ST LV, Dentsply Sirona, Charlotte, NC, USA), the second group (II) was loaded with RMGIC (Fuji II LC CAPSULE, GC Corporation, Tokyo, Japan) and the third group (III) was loaded with GIC (Fuji IX, GC Corporation, Tokyo, Japan). Restorative materials (shade A1) were used in the present study.

### 2.2. Subgroups

Each group was further divided into three subgroups randomly (*n* = 10) as follows: subgroup A, which consisted of sound dentin and immediate loading of the restorative material (control specimens); subgroup B, which consisted of demineralized dentin, SDF treatment and immediate loading of the restorative material; and subgroup C, which consisted of demineralized dentin, SDF treatment and delayed loading of the restorative material a week later.

### 2.3. Application Protocol for the Specimens

A slow speed cutting machine (IsoMet, Buehler, Plymouth, MN, USA) was used to remove the occlusal enamel of the teeth specimens. The complete removal of the enamel was ensured by examining the dentin surfaces of the specimens under a stereomicroscope (SM80, Swift microscope, Carlsbad, CA, USA). The control subgroup specimens for the three groups were loaded immediately with RBC, RMGIC and GIC restorative materials. The material application procedure is described in Table 1. The action of SDF can be verified by its caries arresting property, and in order to simulate dental caries, specimens in subgroups B and C were demineralized. In subgroups B and C, the specimens were demineralized for a period of seven days with pH adjusted to 5.0 (acidic) at 37 °C, as mentioned by Lippert et al. [24].

### 2.4. SDF Treatments

In subgroups B and C for the three groups, the occlusal surface of the demineralized dentin specimens were treated uniformly with one drop of 38% SDF (Advantage Arrest, Elevate Oral Care, West Palm Beach, FL, USA), applied using a microbrush applicator tip. After the SDF application had dried and with no rinsing step, the dentin specimens in subgroup B for the three groups underwent immediate loading with RBC, RMGIC and GIC (5 mm height) using a custom-made mold. Likewise, the specimens in subgroup C for the three groups were treated with SDF similar to subgroup B and were then loaded with RBC, RMGIC and GIC restorative materials, one week later after being stored at 37 °C in distilled water.

### 2.5. µTBS Measurements and Failure Modes

The specimens were sectioned in all groups by a cutting machine with slow speed (IsoMet™, Buehler, Plymouth, MN, USA) to produce 1 mm^2^ specimens and were measured for the μTBS of RBC, RMGIC and GIC by using an Instron testing machine (Instron Corporation, Norwood, MA, USA) with a load of 5 kilo-Newtons with a speed rate of 1 mm/min. The μTBS required to fracture specimens was recorded in megapascal units (MPa). Failure modes were evaluated utilizing a stereomicroscope (SM80, Swift microscope, Carlsbad, CA, USA) and classified as adhesive, cohesive and mixed failures.

### 2.6. Statistical Analysis

Statistical analysis was performed using Statistical Package for Social Sciences (SPSS V.20, IBM Inc., Chicago, IL, USA). The μTBS values were compared using one-way ANOVA and Tukey’s post-hoc tests. The level of significance was set at *p* < 0.05.

## 3. Results

The values of the mean and standard deviation for the µTBS according to the type of restorative material, namely, RBC, RMGIC and GIC, are presented in Table 2. There was significant difference (*p* < 0.05) in the mean µTBS values. RBCs showed the highest µTBS, followed by RMGIC. However, the lowest µTBS value was found with GIC.

Table 3 shows significant differences with regard to the mean µTBS values in each group that comprises the restorative materials and their respective loading times on sound dentin (control group) and demineralized SDF-treated dentin, thus giving more clarity regarding the sampling protocol. The control groups that comprised sound dentin and immediate loading of the restorative materials showed the lowest µTBS when compared to that of the demineralized SDF-treated dentin. Multiple comparisons were assessed, and it was evident that there was an increase in the µTBS in the delayed loading groups, irrespective of the restorative material used. There were no significant differences (*p* > 0.05) in the µTBS with regard to the immediate and delayed loaded groups of RBCs on SDF-treated demineralized dentin, as shown in Table 3. However, multiple comparisons of the µTBS with regard to the immediate and delayed loaded groups of RMGIC on SDF-treated demineralized dentin were significantly different (*p* = 0.049). RBC, RMGIC and GIC loaded in the control group (sound dentin) and delayed loaded group exhibited statistically significant differences (*p* < 0.05).

**Figure 1:**
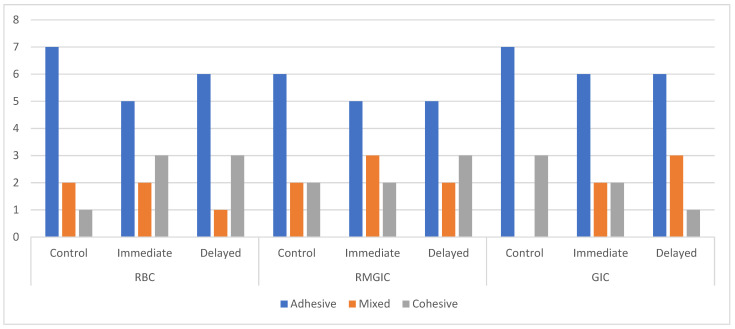
Failure mode distributions for each group. *x* axis: failure modes; *y* axis: number of specimens.

**Figure 2:**
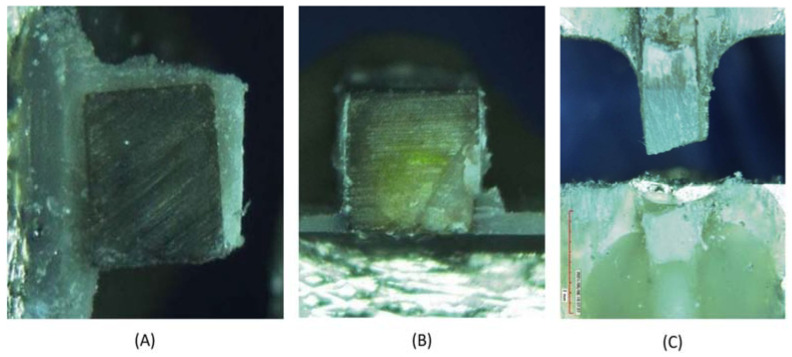
Failure modes of the specimens: adhesive failure (**A**), mixed failure (**B**) and cohesive failure (**C**).

The failure modes for the groups are presented in Figure 1 and Figure 2. All three types of failure modes were identified within the examined specimens. The majority of the failure modes were the adhesive type in the groups. Some cohesive and mixed failures were also noted in the groups. Regarding the mixed failure modes, none were present in the healthy dentin that was loaded with GIC, and only one was present in the delayed group that was loaded with RBCs. Cohesive failures were observed to be greater in SDF-treated groups than in the control group with regard to RBC and RMGIC. The GIC groups showed a greater number of cohesive failure modes for the control group that comprised sound dentin than for the groups with SDF-treated demineralized dentin. No premature failures were noted with the specimens.

## 4. Discussion

The effect of the immediate versus delayed loading time on the μTBS of RBC, RMGIC and GIC restorative materials bonded to demineralized dentin treated with SDF is reported here. Certain limitations of this study must be cautiously considered when interpreting the results. First, as an in vitro study, the actual clinical application of dental materials in a constant moist oral environment with regard to various salivary factors and abundant oral microorganisms may be difficult to implement. Nevertheless, the results of the present study may be considered a valuable contribution to the ongoing SDF studies.

An evaluation of µTBS was conducted in this study rather than using the traditional TBS that tested a larger bonded surface area that generated more adhesive failures and better stress distribution [25]. The correlation between the µTBS and the marginal discoloration of the restorations due to the SDF caries-arresting property and other clinical parameters, such as retention rate, were not taken into consideration, and this may be a direction for future research.

There were four ways the SDF application protocol prior to restorations was implemented in several studies that evaluated the bond strength, namely, rinsing [21,26], air-drying [20,27], polishing [27,28] and application of KI [10,21], to minimize black staining. Due to the inconsistencies in the SDF application protocol, the results of these studies either involved no effect or a decrease in bond strength of adhesive materials on sound dentin or GIC products on SDF- or SDF/KI-treated demineralized dentin. In this study, the SDF application protocol was followed as instructed by the manufacturer. In a clinical situation, the patient is advised to avoid rinsing or consuming food or drinks for a few minutes, although the oral environment is always moist with saliva. Rinsing following SDF application appears to be a crucial step that may improve the bond strength, as suggested by Lutgen et al. [27]. The delayed group had the advantage of being stored for one week in distilled water then the loading of the restorations was performed.

In the present study, RBC exhibited the highest µTBS, followed by RMGIC, and the lowest µTBS was found with GIC. This finding can be correlated to the adhesion mechanism of each of the restorative materials. RBC achieves retention with the help of adhesive systems by either performing micromechanical bonding after etching the dental substrates with phosphoric acid or by ionic/chemical bonding [29]. GIC is an acid-based material that achieves retention by both chemical bonding and micromechanical interlocking. RMGIC, on the other hand, has a polymerizable component, such as 2-hydroxyethyl methacrylate, that helps in better retention [30].

The control groups comprising sound dentin and immediate loading of the restorative materials showed the lowest µTBS when compared to the demineralized SDF-treated dentin. This finding is in accordance with previous studies that claimed that the silver ions from the SDF treatment penetrated deeper into the demineralized dentin than the sound dentin, thereby creating better bonding [31,32]. With regard to the loading time, there was an increase in the µTBS in the delayed loading groups irrespective of the restorative material used. Thus, the null hypothesis was rejected.

An in vitro study by Markham et al. [33] reported that the etching process and universal adhesive bonding of RBCs were negatively affected by contamination with 38% SDF on sound enamel and dentin, owing to its alkaline nature, despite the rinsing step. The authors’ suggestion of employing spot applications with SDF to only the demineralized caries-affected dentin rather than applying it to the entire prepared cavity may be a more beneficial strategy. Taking this into consideration, our study evaluated the µTBS of adhesive restorations loaded at two time intervals on SDF-treated demineralized dentin rather than sound dentin.

The interface between the demineralized dentin, which is highly porous, and the subsequent application of SDF allowing remineralization and covalent bond formation can be very complex [34]. The action of SDF that results in a better resistance of the dental hard tissues may be favorable for the bonding of the various restorative materials [9]. The storage of the SDF-treated demineralized dentin specimens in distilled water for one week influenced the bonding capacity of the restorations in our study.

The greater number of adhesive failure modes noted in this study may be attributed to the poor quality of the hybrid layers that were created between the bonding interfaces, and this result is in accordance with other in vitro studies [19,35]. The authors correlated the other failure modes, such as mixed and cohesive failure modes, to the relatively high bond strength and undeniably weakened demineralized dentin, respectively. In a systematic review, Jiang et al. [15] suggested that the specimens that exhibited cohesive failures should be removed, as this type of failure is caused by inadvertent mechanical errors during the testing process or the brittle nature of the material.

The bonding of restorative materials to dentin depends largely on the application of various heterogenous dental substrates and altered dentinal surfaces [31]. It can be assumed that there may be a failure in the µTBS that could eventually lead to recurrent caries beneath the restorations when they are loaded immediately after the SDF treatment of demineralized dentin is performed. A delay of one week following SDF treatment seemed to obtain better µTBS results in the current study. The structural modification of dentin provoked by SDF treatment over a period of one week may have affected the mechanical properties, such as the µTBS, of the restorative materials. This finding has relevant clinical implications, as immediate loading of the restorations may lead to premature failure, and thus, a delayed approach of loading restorations one week following SDF application may aid in maintaining the advantages of each component. Future studies are needed to assess long-term durability of delayed loading of the restorations on SDF-treated demineralized dentin.

## 5. Conclusions

Within the limitations of this study, the following conclusions were made:

1. The RBC exhibited a greater µTBS than RMGIC and GIC following the application of SDF.
2. Delaying RMGIC loading time for one week following SDF application was observed to have a greater µTBS than that of immediate loading.

The clinical implication of this study is that a delayed loading of the restorations at least one week following SDF application would be better in terms of achieving adequate bond strength.

## Figures and Tables

**Table 1:** Application mode of the restorative materials.

Material	Application Mode
Advantage Arrest, Elevate Oral Care	- Dry the specimen. - Dispense 1 drop of solution into a dappen dish. - Apply the material with an applicator to the tooth surface. - Allow the material to air dry for 5 min without rinsing.
Neo Spectra ST LV, Dentsply Sirona	- Apply 2 mm increments until reaching a 5 mm height. - Cure for 20 s at 1200 mW/cm^2^.
Fuji II LC CAPSULE, GC Corporation	- Apply 2 mm increments until reaching a 5 mm height. - Cure for 20 s at 1200 mW/cm^2^.
Fuji IX, GC Corporation	- Apply 5 mm then let it set for 3 min.
Best-Etch, Vista Dental designs	- Apply to the surface of the bonding for 15 s. - Rinse with water for 5 s and dry.
Cavity conditioner, GC Corporation	- Apply to the bonding surfaces for 10 s. - Rinse with water for 5 s, then dry.
Prime & Bond NT, Dentsply Sirona	- Apply for 20 s using a microbrush. - Air dry gently for 5 s. - Cure for 10 s at 1200 mW/cm^2^.

**Table 2:** The mean and standard deviation (SD) values of the microtensile bond strength in megapascal (MPa) based on the type of each group.

Group	Total N = 90 *n*	Mean ± SD	*p* Value	Multiple Comparison Test		
				RBC	RMGIC	GIC
RBC	30	24.44 ± 3.76	<0.001 *	1	<0.001 *	<0.001 *
RMGIC	30	13.96 ± 3.69		<0.001 *	1	<0.001 *
GIC	30	5.34 ± 2.22		<0.001 *	<0.001 *	1

* Significantly different as tested by one-way ANOVA and post-hoc Tukey tests.

**Table 3:** The mean and standard deviation (SD) values of the microtensile bond strength in megapascal (MPa) based on loading time of each group.

Group	Loading Time	Total N = 90 *n*	Mean ± SD	*p* Value	Multiple Comparison Test		
					Control	Immediate	Delayed
RBC	Control	10	21.26 ± 2.92	<0.001 *	1	0.027 *	<0.001 *
	Immediate	10	24.88 ± 3.01		0.027 *	1	0.209
	Delayed	10	27.17 ± 2.87		<0.001 *	0.209	1
RMGIC	Control	10	11.53 ± 3.76	0.002 *	1	0.335	0.002 *
	Immediate	10	13.49 ± 2.72		0.335	1	0.049 *
	Delayed	10	16.86 ± 2.50		0.002 *	0.049 *	1
GIC	Control	10	3.83 ± 1.87	0.022 *	1	0.090	0.023 *
	Immediate	10	5.81 ± 2.12		0.090	1	0.806
	Delayed	10	6.37 ± 2.02		0.023 *	0.806	1

* Significantly different as tested by one-way ANOVA and post-hoc Tukey tests.

## Data Availability

Data are available upon request.
